# Thematic Maps of the Impact of Urbanization and Socioeconomic Factors on the Distribution of the Incidence of Cutaneous Leishmaniasis Cases in Sefrou Province, Central North of Morocco (2007–2011)

**DOI:** 10.1155/2020/8673091

**Published:** 2020-07-14

**Authors:** Fatima Zahra Talbi, Nordine Nouayti, Hajar El Omari, Mohamed Najy, Khadija Lahouiti, Mouhcine Fadil, Hassan Ech-Chafay, Mohamed Lachhab, Abdellatif Janati Idrissi, Abdelhakim El Ouali Lalami

**Affiliations:** ^1^Laboratory of Biotechnology and Preservation of Natural Resources, Faculty of Sciences Dhar El Mahraz, Sidi Mohamed Ben Abdellah University, Fez 30000, Morocco; ^2^Applied Sciences Laboratory, Water and Environmental Engineering Team, National School of Applied Sciences, Abdelmalek Essaadi University, Tetouan, Morocco; ^3^Natural Resources Management and Development Team, Laboratory of Health and Environment, Faculty of Sciences, Moulay Ismail University, Meknes, Morocco; ^4^Laboratory of Agrophysiology, Biotechnology, Environment and Quality, Department of Biology, University Ibn Tofail, Faculty of Science, BP 133, Kenitra 14000, Morocco; ^5^Laboratory of Microbial Biotechnology, Department of Biology, Faculty of Sciences and Technology, University Sidi Mohamed Ben Abdellah, BP 2202, Road of Immouzer, Fez, Morocco; ^6^Physico-chemical Laboratory of Inorganic and Organic Materials, Materials Science Center (MSC), Ecole Normale Supérieure, Mohammed V University in Rabat, Rabat, Morocco; ^7^Higher Institute of Nursing Professions and Health Techniques of Fez, Regional Health Directorate Fez-Meknes, EL Ghassani Hospital, Fez 30000, Morocco

## Abstract

**Background:**

Leishmaniases are vector-borne diseases with health risks. They cause a big health problem. These parasitic diseases are transmitted by the parasite of the genus *Leishmania* through sandflies.

**Objective:**

The aim of this work is to study the distribution of the incidence of cutaneous leishmaniasis (CL) cases and the impact of urbanization and socioeconomic factors and their effects as leishmaniasis risk factors.

**Methods:**

We conducted a retrospective study of CL cases collected at the level of Sefrou Province during the period from 2007 to 2011. The data was collected from registers of the Medical Delegation of Sefrou Province. The socioeconomic data, namely, the poverty rate, the popular density, and the type of environment (urban/rural) of Sefrou Province, were obtained from the High Commission for Planning. Statistical analysis was performed by SPSS software (version 20). The data were registered in a Microsoft Excel 2010 file. Statistical analysis was based on one-way analysis of variance (ANOVA), and then a correlation study was carried out (Pearson correlation). The results were considered significant when *p* was less than 0.05. The database was analyzed by QGIS 2.18, which is open source software.

**Results:**

A total of 349 cases of CL were collected at Sefrou Province from 2007 to 2011. A percentage of 49% of the cases come from urban areas, while 51% of the cases come from rural areas. In the statistical analysis, the division of the incidence of CL cases was found to be significantly associated only with urbanization. For the other factors, the number of people or the poverty rate is not taken into account in the incidence dynamics.

**Conclusion:**

This study may be useful for the implementation of future adequate measures and controls. Getting rid of leishmaniasis requires a comprehensive approach by acting on the sources of contamination through good continuous surveillance, appropriate management, effective vector control, and awareness-raising strategies.

## 1. Introduction

Leishmaniasis is a vector-borne disease caused by a flagellated protozoan of the genus *Leishmania* belonging to the family of Trypanosomatidae, which affects a variety of human and animal populations worldwide [1]. The epidemiology of leishmaniasis is very complex: 20 species of *Leishmania* are pathogenic to humans, and 30 species of sandflies are proven vectors in the world [2]. It is widespread in all continents with the exception of Oceania. Eighty-eight countries are affected in the Old World and twenty-two countries in the New World [3]. The global prevalence is estimated at 12 million human cases, with an annual incidence of 1.5 to 2 million new cases, and 350 million people would be exposed to the risk of *Leishmania* transmission [4]. This alarming situation is of paramount importance since *Leishmania*/HIV coinfections are frequent, and immunocompromised patients are much less controlled, which increases mortality [5]. In Morocco, leishmaniasis is endemic; the Ministry of Health has declared an annual average of 4,076.6 cases of cutaneous leishmaniasis (CL) and a hundred cases of visceral leishmaniasis (VL) in recent years [6]. In the majority of countries around the Mediterranean, leishmaniasis diseases represent entities of great clinical and epidemiological diversity and constitute a real health problem [7]. They can be illustrated in three epidemiological entities: zoonotic cutaneous leishmaniasis (ZCL) with *Leishmania major* (*L. major*), anthroponotic cutaneous leishmaniasis (ACL) with *Leishmania tropica* (*L. tropica*), and VL with *Leishmania infantum* (*L. infantum)* [8]. The geographical position of Morocco is characterized by different ecological and bioclimatic conditions which can influence the diversity of vectors and consequently the distribution of the disease [9]. The choice of Province Sefrou as a study area is justified by its epidemic situation of leishmaniasis, as it is considered among the main foci, by means of its proximity to other important foci such as Taza [10], Sidi Kacem [11], and Errachidia [12]. Several entomological studies were pursued at the Province Sefrou that shows the presence of sandflies with a significant density [13–15]. The spread of cutaneous leishmaniasis disease is linked to various risk factors [16]; among these factors we notice the socioeconomic factors and the urbanization ones [17, 18]. For several years, risk mapping has been used for several researches based on remote sensing and Geographic Information Systems (GIS). Indeed, these tools are of supreme interest with regard to epidemiological surveillance and the relationship between the spread of the disease and environmental factors [19–21]. For an effective control, it is necessary to establish a risk map of CL from different municipalities in Sefrou Province. This work is a combination of the retrospective study and the risk factors for the distribution of CL: climate, urbanization, poverty, and population. The use of GIS allows us to draw up thematic maps identifying areas at risk. Cartographic analysis is very important to better elucidate the relationship between the distribution of CL disease and the factors of leishmaniasis risk. The aim of this present work is to study the epidemiological situation of this type of parasitosis, the distribution of the incidence, and the impact of urbanization and socioeconomic factors as leishmaniasis risk factors.

## 2. Materials and Methods

### 2.1. Study Zone

Sefrou Province (Central North of Morocco) covers an area of 3,520 km^2^ with a predominantly rural population of 259,254 inhabitants. From a geographical point of view, it is limited to the south by the Boulemane and Ifrane Provinces, to the east by the Taza Province, to the north by the Fez prefecture, to the west by the Moulay Yacoub and El Hajeb Provinces, and to the northwest by the Taounate Province [22]. According to the municipal division, Sefrou Province has 5 municipalities and 18 rural communes. Most of these mountainous provinces are contaminated and considered as places of human CL [23].

### 2.2. Data Sources

The data were collected from the registers of the health directorate of Sefrou Province. All cases of CL recorded on the survey forms were confirmed by parasitological diagnosis direct. CL patients with clinical lesions of CL were passively received during the study period at the local laboratory of the health center of Sefrou Province. [24]. The period of the study was four years, from 2007 to 2011. During this period, a total of 349 cases of CL were the subjects of a retrospective study. The socioeconomic data, namely, the poverty rate, the popular density, and the type of environment (urban/rural) of Sefrou Province, were obtained from the High Commission for Planning [25]. The incidence (I) of CL of each commune was calculated as follows:

(1) $$\begin{array}{c} I=\frac{\text{number of new cases of the disease in a given population}}{\text{total number of the population at risk of each commune}}. \end{array}$$

### 2.3. Data Analysis

The data were registered in a Microsoft Excel 2010 file. Statistical analysis was based on one-way analysis of variance (ANOVA), and then the correlation study was carried out (Pearson correlation). The results were considered significant when *p* is less than 0.05. To achieve the objective of this study, it is essential to integrate the CL health data into a Geographic Information System in order to analyze its spatial distribution in the various municipalities of the Province of Sefrou. In this respect, the data processing was done by QGIS 2.18 software by the integration of Geographic Information System. Statistical analysis was performed with SPSS software (version 20).

## 3. Results and Discussion

### 3.1. Epidemiology of Leishmaniasis in Sefrou Province

**Figure 1:**
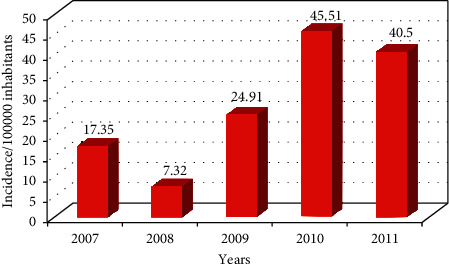
Temporal evolution of the annual incidence of CL cases/100,000 inhabitants in Sefrou Province (2007–2011).

The temporary evolution of the incidence of CL revealed the endemic nature of this parasitosis in Sefrou Province. The study of the epidemiological situation in this Province during the period from 2007 to 2011 declared 349 cases of CL with an average of 5.77 per 100,000 inhabitants. In 2007, the incidence of CL cases was 17.35 per 100,000 population. This value decreased in 2008 to 10.03. From 2009, the incidence of cases increased with a peak in 2010 and decreased afterward (Figure 1).

This could be explained by the fact that the situation of this disease has become perturbing, which is linked to the appearance of new foci of transmission where the risk factors persist [16, 26, 27]. Also, the alarming epidemiological situation in 2010 witnessed better management of this public health problem, good awareness, and therefore greater treatment of patients. A cartographic study, on the same province, was able to show that during this year the rural areas were affected by a high rate of incidence (rural municipality of Tazouta and Ahl Sidi Lahcen) [16]. This result corroborates that of the Meknes Province [28] where the incidence varies in time and space; during the same period from 2009 to 2013, the incidence experienced a very marked development, which is justified by unhygienic environmental conditions which increase the risk of leishmaniasis by promoting the multiplication of vectors.

Statistical analysis of this result shows that there is no relationship between the distribution of cases and time (the *p* value is greater than 0.05).

### 3.2. The Impact of the Urbanization Factor on CL Disease in Sefrou Province

According to the administrative division, Sefrou Province is made up of 24 communes, divided into 7 urban communes and 17 rural communes (Table 1) [25].

**Figure 2:**
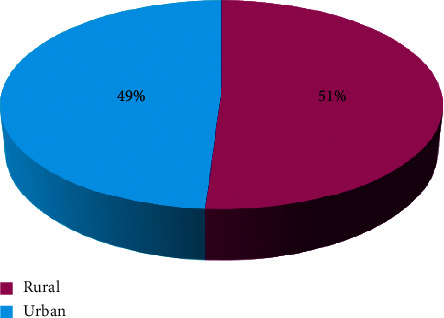
Distribution of the incidence of CL cases according to the state of urbanization of the municipalities of Sefrou Province (2007–2011).

According to the census of CL cases, the disease occurs with high incidence in municipalities more than others. We found 49% of recorded cases of urban origin (Figure 2) with a maximum incidence at El Menzel commune of 66.28 per 10,000 inhabitants. For rural municipalities, 51% of cases (177 cases) were reported with a maximum incidence at the level of Azzaba commune with 76.21 per 10,000 inhabitants, followed by Tazouta commune (74.84 per 10,000 inhabitants) and that of Ahl Sidi Lahcen (66.16 per 10,000 inhabitants).

The analysis of variance revealed a statistically significant difference between the medium numbers of cases from one level of urbanization to another with a confidence rate of 95.0% (df = 1, *F* = 4.73, *p*=0.041) Table 2. Accordingly, there is a significant effect between these two variables with a decision of 4.73%.

According to the literature, urbanization interests all continents and all countries [29]. This factor is closely linked to the massive migration to urban areas specifically in the outskirts of cities where there is a lack of waste management, which increases the reproduction of sandflies. In addition, urban CL of the Old World characterizes the anthroponotic type where transmission is carried out through a female sandfly infected with epidermis from one subject to another. This epidemic character is strictly linked to the density of sandflies and the human population. In addition, according to the World Health Organization [30], the phenomenon of urbanization increases the risk of transmission of leishmaniasis especially within an unimmunized population in rural areas. The lack of planning of cities in rural exodus and poor environmental hygiene conditions [31] increase the leishmaniasis risk and lead to favorable bioecological environments to the development of vectors [32–34]. Several studies reveal that urbanization contributes to the increase in the incidence of ACL type [35, 36]. They also confirmed that the rural areas, where most leishmaniasis patients live, coexist in interresidential homes, or occupy dwellings, allow contact between humans and sandflies and subsequently ensure the circulation of the parasite [27, 37].

### 3.3. The Impact of Socioeconomic Factors in Different Municipalities in Sefrou Province on the Distribution of the Incidence of CL Cases

#### 3.3.1. Distribution of the Incidence of CL Cases according to the Population Factor of Sefrou Province

According to the population census of Sefrou Province [25], the number of inhabitants is 259,254 (Table 3).

**Figure 3:**
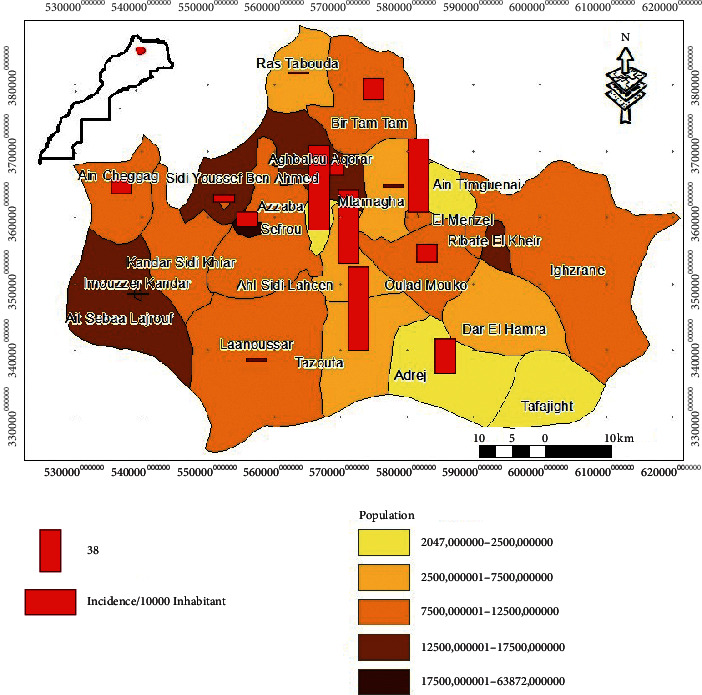
Distribution map of the incidence of CL cases of inhabitants at the level of different municipalities in Sefrou Province (2007–2011).

The distribution of the incidence of CL according to the number of inhabitants of each municipality of Sefrou Province (Figure 3) shows that the municipalities with high incidence rate (Azzaba, Tazouta, El Menzel, and Ahl Sidi Lahcen) have a moderately small number of population.

This distribution of the incidence is not the same at the level of the other communes with low number of population where the incidence rate is low such as commune Ras Tabouda, Bir Tam Tam, and Bhalil. On the other hand, conversely, the commune of Sefrou with its overcrowded situation has been reported to have moderately low incidence (only 13.62 per 10,000 inhabitants) (Figure 3).

According to our result, the majority of municipalities with a high incidence rate are not overcrowded and of rural origin. In addition to the small number of population, the environmental characteristics of these environments make the situation more alarming, which leads to rapid dispersion of cases. Statistical analysis revealed that the distribution of the incidence of CL in this province is not linked to the number of inhabitants by municipality, but to the percentage of cases (Table 4). A positive correlation of 0.474 was confirmed by the normality tests of Kolmogorov–Smirnov (*p*=0.003 < to 0.05) and Shapiro–Wilk (*p*=0.001 < to 0.05), which reveals that the studied samples follow a normal distribution.

From 2007 to 2011, we observed that the number of cases increased with a peak in 2010. This peak could explain the success of the initiative and the management of the disease by the ministry and various authorities. The objectives of this program aim primarily to raise awareness among the population as well as intensifying mass screening activities in schools and localities at risk, following the recommendations of the response plan. Therefore, the mentioned evolution of cases could be explained by the implementation of the strategy for the fight against this disease and the information campaigns conducted by the Ministry of Health according to the national plan for the fight against leishmaniasis, launched in 2009 (Program of Integrated Management of Vector Control). In addition, the factor of the increasing density of the population and the propagation of favorable milieus to the multiplication of the vector could also have an effect on this situation [38]. Indeed, Old World anthroponotic cutaneous leishmaniasis (ACL) requires a reservoir which is humans [15]. This means that the parasite is transmitted from one subject to another through the bite of a peridomestic sandfly, once the infected subjects arrive to the endemic area. Thereby, this can contribute to the extension of its geographical area. We can also explain that the municipalities which have several geographic (proximity to Fez) and economic advantages are highly attractive municipalities. In case of a contamination, overcrowd is directly associated with the high number of CL cases.

#### 3.3.2. Distribution of the Incidence of CL Cases according to the Poverty Factor of Sefrou Province

**Figure 4:**
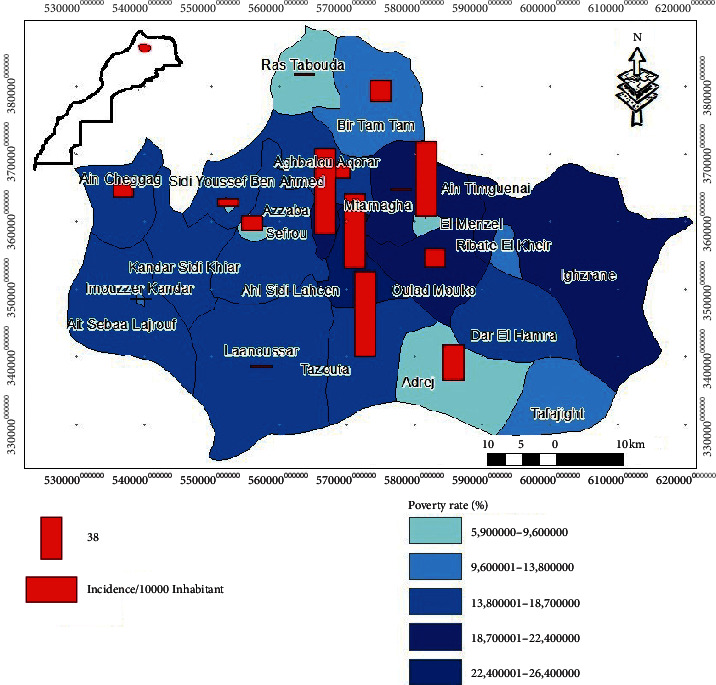
Distribution map of the incidence of CL cases according to poverty factor at the level of different municipalities Sefrou Province (2007–2011).

Figure 4 shows the distribution of the incidence of CL cases according to the poverty rate of different municipalities in Sefrou Province. The poverty rate varies from 5.9% to 26.4% according to data from the High Commission of Planning.

According to the descriptive analysis, it appears that the incidence of CL does not depend on the poverty rate. Indeed, at the level of the communes of Azzaba (76.21 per 10,000 inhabitants) and Tazouta (74.84 per 10,000 inhabitants), where the incidence is very high compared to the other communes, the poverty rate is ordered, respectively, as 20% and 17.5%, whereas at the level of the commune of El Menzel, poverty was only 9.6% despite the high incidence rate of 66.28 per 10,000 inhabitants (Figure 4).

The statistical analysis of this result shows that there is no link between the distribution of the incidence of CL cases and the poverty rate at the level of Sefrou Province; this is justified by the *p* value with the order 0.55 which is greater than the significance level of 5% with a relatively very low correlation coefficient (*p* > 0.05, *r* = 0.13) (Table 5).

According to the literature, socioeconomic factors such as poverty and lack of infrastructure seem to be among the main factors that lead to leishmaniasis [39, 40]. In endemic areas, the leishmaniasis risk is strongly linked to the bad hygienic conditions of the environment [3]. Internal migration of the population for difficult economic reasons led to the establishment of peripheral areas of cities, which leads to favorable environments to the rapid spread of vectors. In addition, poverty rate is an indicator that entails bad nutrition, causing population to be more amenable to the pathogen [41]. Another study has shown that poverty can increase the risk of leishmaniasis due to poor hygienic conditions [41]. In our case, we were able to show another result; that is, poverty does not influence the distribution of the incidence of CL cases at the level of Sefrou Province but has an impact on the distribution of CL cases. A study in Quebec coincides with ours and it has been able to show that poverty does not explain the emergence of CL [42]. The same is true in Morocco, especially in Meknes Province, as the poverty rate of municipalities has no influence on the distribution of the epidemic [11].

## 4. Conclusion

Various factors can have an effect on the distribution of leishmaniasis. This work emphasizes the effect of urbanization and socioeconomic risks on the distribution of cases of cutaneous leishmaniasis at the level of Sefrou Province. The use of GIS is an added value to the creation of thematic maps which function as CL risk maps. These results must be taken into consideration in determining the risk zones for leishmaniasis and their risk factors in order to establish effective control strategies.

## Figures and Tables

**Table 1:** Distribution of the incidence of CL cases according to the urbanization factor.

Commune	Incidence/10,000 inhabitant	Type of urbanization	Total
Adrej	31.3	Rural	177 cases (51%)
Aghbalou Aqorar	13.26	Rural	
Ahl Sidi Lahcen	66.16	Rural	
Ain Cheggag	15.39	Rural	
Ain Cheggag	0	Rural	
Ain Timguenai	0	Rural	
Ait Sebaa Lajrouf	0	Rural	
Azzaba	76.21	Rural	
Bir Tam Tam	18.52	Rural	
Dar El Hamra	0	Rural	
Ighzrane	0	Rural	
Kandar Sidi Khiar	0	Rural	
Laanoussar	2.14	Rural	
Mtarnagha	1.89	Rural	
Oulad Mkoudou	16.62	Rural	
Ras Tabouda	1.53	Rural	
Sidi Youssef Ben Ahmed	0	Rural	
Tafajight	0	Rural	
Tazouta	74.84	Rural	

Bhalil	6.87	Urbane	172 cases (49%)
El Menzel	66.28	Urbane	
Imouzzer Kandar	0.72	Urbane	
Ribate El Kheir	0	Urbane	
Sefrou	13.62	Urbane	

**Table 2:** Test of analysis of variance.

			Sum of squares	df	Medium square	*F*	Significance
Number of CL cases ^*∗*^urbanization	Intergroup	Combined	2,361.604	1	2,361.604	4.739	0.041
	Intraclass		10,465.700	21	498.367		
	Total		12,827.304	22		

Percentage of CL cases ^*∗*^urbanization	Intergroup	Combined	9.415	1	9.415	0.192	0.666
	Intraclass		1,030.145	21	49.055		
	Total		1,039.560	22			

^*∗*^Correlation is significant at level 0.05, df: the degrees of freedom in the source; F: the *F*-statistic.

**Table 3:** Distribution of inhabitants at the level of different municipalities in Sefrou Province (2007–2011).

Commune	Population
Adrej	2,236
Aghbalou Aqorar	15,835
Ahl Sidi Lahcen	5,290
Ain Cheggag	11,039
Ain Cheggag	4,286
Ain Timguenai	2,208
Ait Sebaa Lajrouf	17,400
Azzaba	2,493
Bhalil	11,638
Bir Tam Tam	9,714
Dar El Hamra	4,022
El Menzel	11,465
Ighzrane	11,050
Imouzzer Kandar	13,725
Kandar Sidi Khiar	8,709
Laanoussar	9,343
Mtarnagha	5,284
Oulad Mkoudou	7,821
Ras Tabouda	6,516
Ribate El Kheir	12,654
Sefrou	63,872
Sidi Youssef Ben Ahmed	11,292
Tafajight	2,047
Tazouta	5,745
Zaouia Bougrine	3,570
Total	259,254

**Table 4:** Pearson correlation and significance between incidence of CL cases and the population factor.

		Population	Percentage of CL cases
Incidence per 10,000 population	Pearson correlation	−0.135	0.474^*∗*^
	Sig. (bilateral)	0.540	0.022
	*N*	23	23

^*∗*^Correlation is significant at level 0.05 (bilateral).

**Table 5:** Pearson correlation and significance between incidence of CL cases and poverty rate.

		Poverty rate %	Percentage of CL cases
Incidence per 10,000 population	Pearson correlation	0.131	0.474^*∗*^
	Sig. (bilateral)	0.552	0.022
	*N*	23	23

^*∗*^Correlation is significant at level 0.05 (bilateral).

## Data Availability

No data were used to support this study.
